# Aging impairs peritoneal but not bone marrow-derived macrophage phagocytosis

**DOI:** 10.1111/acel.12223

**Published:** 2014-05-12

**Authors:** Eimear Linehan, Yvonne Dombrowski, Rachel Snoddy, Padraic G Fallon, Adrien Kissenpfennig, Denise C Fitzgerald

**Affiliations:** 1 Centre for Infection and Immunity, Queen’s University Belfast97 Lisburn Road, Belfast, BT9 7AE, Northern Ireland; 2 Institute of Molecular Medicine, St. James’s Hospital, Trinity College DublinDublin 2, Ireland; 3 National Children’s Research Centre, Our Lady’s Children’s Hospital CrumlinDublin 8, Ireland

**Keywords:** aging, bone marrow, immunity, macrophage, peritoneum, phagocytosis

## Abstract

Aging results in deterioration of the immune system, which is associated with increased susceptibility to infection and impaired wound healing in the elderly. Phagocytosis is an essential process in both wound healing and immune defence. As such, age-related impairments in phagocytosis impact on the health of the elderly population. Phagocytic efficiency in peritoneal macrophages, bone marrow-derived macrophages and bone marrow monocytes from young and old mice was investigated. Aging significantly impaired phagocytosis by peritoneal macrophages, both *in vitro* and *in vivo*. However, bone marrow-derived macrophages and bone marrow monocytes did not exhibit age-related impairments in phagocytosis, suggesting no intrinsic defect in these cells. We sought to investigate underlying mechanisms in age-related impairments in phagocytosis by peritoneal macrophages. We hypothesized that microenvironmental factors in the peritoneum of old mice impaired macrophage phagocytosis. Indeed, macrophages from young mice injected into the peritoneum of old mice exhibited impaired phagocytosis. Proportions of peritoneal immune cells were characterized, and striking increases in numbers of T cells, B1 and B2 cells were observed in the peritoneum of old mice compared with young mice. In addition, B cell-derived IL-10 was increased in resting and LPS-activated peritoneal cell cultures from old mice. These data demonstrate that aging impairs phagocytosis by tissue-resident peritoneal macrophages, but not by bone marrow-derived macrophages/monocytes, and suggest that age-related defects in macrophage phagocytosis may be due to extrinsic factors in the tissue microenvironment. As such, defects may be reversible and macrophages could be targeted therapeutically in order to boost immune function in the elderly.

## Introduction

A principle hallmark of aging is the impairment in immune responses, referred to as immunosenescence. Aging is associated with deterioration of both innate and adaptive immune responses, which contributes to increased susceptibility to infections, decreased vaccine responses and impaired wound healing in the elderly (Linton & Dorshkind, 2004; Gomez *et al*., 2008; McElhaney & Effros, 2009; Mahbub *et al*., 2011; Montecino-Rodriguez *et al*., 2013). The investigation and understanding of immune aging is becoming an increasingly important area of research due to the advancing average age of the global population and the clinical challenges in treating elderly patients (Dorshkind *et al*., 2009).

Macrophages play a critical role in immunity and represent a first line of defence against invading pathogens. Macrophages are potent phagocytic and highly versatile cells, capable of antigen presentation and secretion of a wide range of factors including cytokines and chemokines (Gordon & Taylor, 2005). Many studies have reported that macrophage function is impaired with aging (Plowden *et al*., 2004; Sebastian *et al*., 2005; Shaw *et al*., 2010). Decreased pro-inflammatory cytokine secretion by macrophages in response to TLR stimulation has been demonstrated during aging in rodents (Renshaw *et al*., 2002; Boehmer *et al*., 2004; Chelvarajan *et al*., 2005), and responsiveness to IFN-γ is also diminished with age (Ding *et al*., 1994; Yoon *et al*., 2004). In addition, MHC class II expression is reduced in macrophages from old mice compared with young mice (Herrero *et al*., 2001).

Phagocytosis is an essential process in defence against pathogens, clearance of apoptotic cells and wound healing. As such, age-related impairments in phagocytosis are likely to contribute to susceptibility to infection and impaired wound healing. However, literature regarding the impact of aging on macrophage phagocytic function is conflicted, and apparently opposing results have been reported. De La Fuente demonstrated a decrease in macrophage phagocytosis in old mice compared with young mice (De La Fuente, 1985). However, while an age-related decline in phagocytosis by neutrophils was observed in rats, phagocytosis by alveolar macrophages was not impaired by age in the same model (Mancuso *et al*., 2001). Aprahamian *et al*. demonstrated an age-related reduction in phagocytosis of apoptotic cell debris in mice *in vivo* but not *in vitro* (Aprahamian *et al*., 2008). Microglia isolated from old mice internalized less amyloid beta peptide compared with microglia isolated from young mice *in vitro* (Njie *et al*., 2012); however, others have observed an age-related increase in phagocytic activity in microglia from rats (Lynch *et al*., 2010). Macrophages from wounds in old mice exhibited impaired phagocytic activity, resulting in delayed removal of debris (Swift *et al*., 2001). Furthermore, recent human studies have reported impaired phagocytosis by CD14^+^ monocytes from older individuals (Hearps *et al*., 2012), while earlier studies reported no effect of aging on phagocytic function in humans (Fietta *et al*., 1994).

There are two main theories regarding underlying mechanisms involved in age-related impairments in macrophage function in general. One hypothesis proposes that aging results in intrinsic defects in macrophages. For example, impaired intracellular signalling has been reported in response to IFN-γ (Yoon *et al*., 2004). An alternative hypothesis proposes that factors in the microenvironment of old mice affect macrophage function (Chen *et al*., 1996; Mahbub *et al*., 2012). It has been established that macrophages are highly plastic and can readily adapt to changes in environmental stimuli (Stout & Suttles, 2004). Further studies are required to elucidate the reasons for age-related impairments in macrophage function, where impairment has been clearly demonstrated. The ultimate goal of immune aging research is to provide therapies to boost immune responses in the elderly. It is essential to establish the underlying mechanisms of age-related macrophage impairment, in order to aid the design of treatments to modulate macrophage function.

In this study, the impact of aging on macrophage phagocytosis was comprehensively investigated in C57BL/6 mice. Macrophages from different tissue sites were studied, and both *in vitro* and *in vivo* assays were performed. We observed that aging significantly decreased phagocytosis of fluorescent particles by peritoneal macrophages. However, this age-related impairment in phagocytosis was not observed in bone marrow-derived macrophages (BMDMs) or monocytes isolated directly from bone marrow of old mice, indicating no intrinsic defect in phagocytosis in macrophage progenitors in this model. We hypothesized that factors in the peritoneum of old mice downregulated macrophage function. Indeed, macrophages from young mice, injected into the peritoneum of old mice, showed reduced phagocytosis. This could be due to the significantly increased population of both B1 and B2 cells and increased IL-10 responses that were observed in the peritoneum of old mice. This study provides insight into the age-related impairment in macrophage phagocytosis, and these findings could be important for the development of immune-boosting therapies for the elderly.

## Results

### Aging impairs phagocytosis by peritoneal macrophages

**Figure 1:**
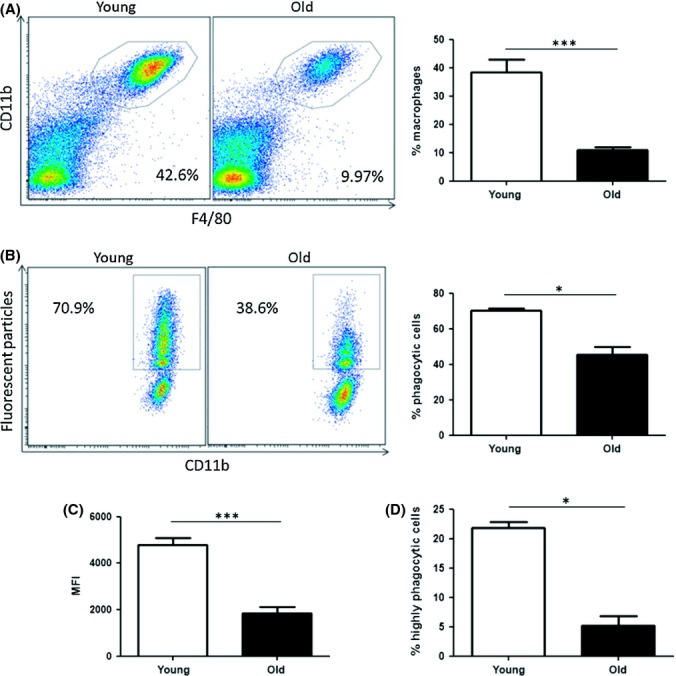
Phagocytosis of fluorescent particles is reduced in peritoneal macrophages from old mice. Percentage of CD11b^+^/F4/80^+^ positive cells was assessed in peritoneal cells from young and old mice by flow cytometry. Representative dot plot and data combined from *n* = 8 mice (A). Total peritoneal cells were harvested from young and old mice, and macrophages were purified using an immunomagnetic negative selection kit. Macrophages were incubated with fluorescent particles (50 particles/cell) for 2 h and phagocytosis was analysed by flow cytometry (B). Mean fluorescence intensity (MFI) values were calculated (C), and the percentage of highly phagocytic macrophages in cultures from young and old mice was assessed (D). One representative experiment shown of three experiments, *n* = 4 per experiment. Mann–Whitney tests were performed for nonparametric data. Unpaired Student’s *t*-tests were performed for parametric data. *P < 0.05 *** *P* < 0.001.

To investigate the phagocytic capacity of macrophages from young (8–12 weeks) and old (15–20 months) mice, purification of peritoneal macrophages was first performed. This purification step was essential as we found that the percentage of macrophages in the peritoneum decreased with age (Fig. 1A), which could lead to misinterpretation of results from nonpurified macrophage cultures. Purification of macrophages, using a novel negative selection monocyte enrichment kit, consistently resulted in cultures of 95–98% CD11b^+^F4/80^+^ double-positive cells (Fig. S1A). Fluorescent particles were incubated with purified peritoneal macrophages from both young and old mice, and phagocytosis was measured by flow cytometry. Phagocytosis was significantly reduced in macrophages isolated from old mice compared with young mice. This was evident in the percentage of macrophages that had taken up fluorescent particles (Fig. 1B). Furthermore, analysis of mean fluorescence intensity, representing mean number of particles taken up by each macrophage, showed a striking and highly significant difference (Fig. 1C), which highlighted the inefficiency of phagocytosis by macrophages from old mice. Indeed, the percentage of highly phagocytic macrophages, engulfing a large amount of particles per cell, was greatly reduced in macrophage cultures from old mice (Fig. 1D, Fig. S1B).

**Figure 2:**
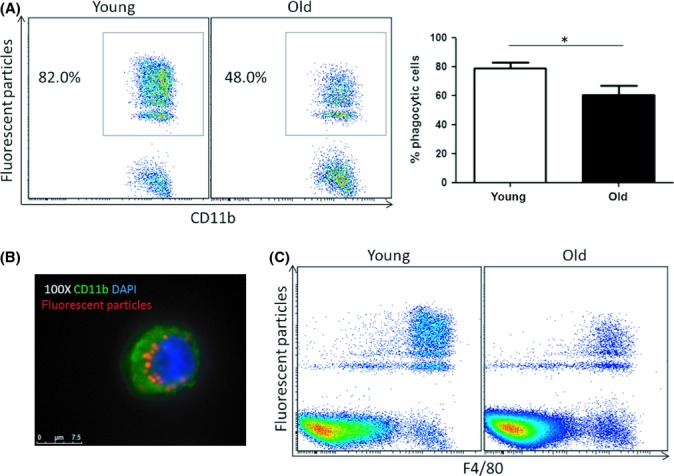
*In vivo* phagocytosis by peritoneal macrophages is reduced in old mice. Fluorescent particles were injected into the peritoneum of young and old mice. Mice were euthanized and peritoneal cells were collected 2 h later. Phagocytosis by peritoneal macrophages was analysed by flow cytometry (A). Internalization of particles was confirmed by immunofluorescence (B). Analysis of flow cytometry data demonstrated that fluorescent particles were predominantly taken up by macrophages *in vivo* (C). *n* = 6 animals per group pooled from three independent experiments. Mann–Whitney tests were performed *P < 0.05.

To determine whether this *in vitro* observation was relevant *in vivo*, fluorescent particles were injected into the peritoneum of young and old mice. Total peritoneal cells were harvested after 2 h to assess *in vivo* phagocytic capacity. Again, there was a consistent reduction in phagocytosis by macrophages *in vivo* in old mice compared with macrophages in young mice (Fig. 2A). In order to confirm that fluorescent particles had been internalized and not merely adhered to the cell surface, cells were stained with DAPI and anti-CD11b FITC and immunofluorescence images were acquired. Fluorescent particles were located within the macrophages (Fig. 2B). Further analysis of flow cytometry data demonstrated that fluorescent particles were taken up by predominantly F4/80^+^ macrophages *in vivo* and not by other cells types in the peritoneum (Fig. 2C).

### Bone marrow-derived macrophages, differentiated *in vitro*, do not exhibit age-related impairments in phagocytosis

**Figure 3:**
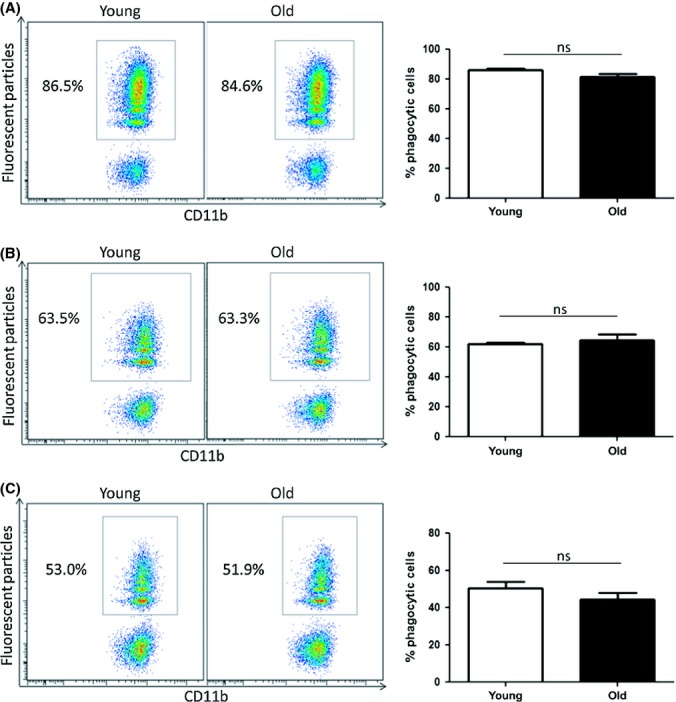
Bone marrow-derived macrophages do not exhibit age-related impairments in phagocytosis. Bone marrow was harvested from young and old mice and cultured for 7 days in the presence of 15% conditioned media from L929 fibroblasts. BMDMs were incubated with fluorescent particles at a concentration of either 50 particles/cell (A and C) or 25 particles/cell (B). Macrophages were harvested after 2 h (A and B) or 30 min (C), and phagocytosis was assessed by flow cytometry. One representative experiment shown of three experiments, *n* = 4 per experiment. Mann–Whitney tests were performed. ns = nonsignificant.

BMDMs are commonly used for *in vitro* macrophage studies. These cells are generated from bone marrow progenitors and are frequently cultured in the presence of conditioned medium from the L929 fibroblast cell line (Weischenfeldt & Porse, 2008). It has been reported that the effect of aging on macrophage function can vary depending on the tissue that macrophages have been isolated from and stimulants that macrophages have been exposed to (Kohut *et al*., 2004). Therefore, macrophages were generated *in vitro* from bone marrow from young and old mice to explore the impact of aging on this artificially differentiated and widely used macrophage population. Fluorescent particles were added to BMDMs for 2 h, and phagocytosis was assessed by flow cytometry. Interestingly, BMDMs from young and old mice consistently showed similar levels of phagocytosis of fluorescent particles (Fig. 3A). This finding was confirmed by performing additional phagocytosis assays with a reduced concentration of fluorescent particles (25 particles/cell) and also a reduced incubation time (30 min) in order to clarify that the system was not saturated. As expected, these changes resulted in decreased levels of phagocytosis. However, in both cases, BMDMs did not show significant age-related impairments in phagocytosis (Fig. 3B,C respectively). Furthermore, analysis of mean particles taken up per cell and percentage of highly phagocytic cells were all similar in BMDM cultures from young and old mice (data not shown). This demonstrates that BMDM generated *in vitro* is not a suitable model for investigating the impact of aging on tissue macrophages such as peritoneal macrophages.

### Bone marrow monocytes do not exhibit age-related impairments in phagocytosis

**Figure 4:**
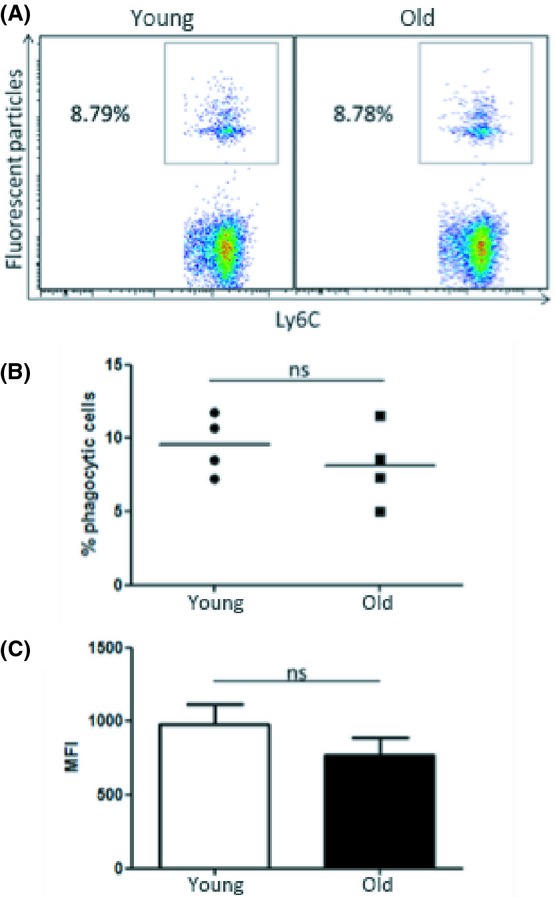
Bone marrow monocytes do not exhibit age-related impairments in phagocytosis. Bone marrow was harvested from young and old mice, and monocytes were isolated from total bone marrow cells using a monocyte enrichment kit. Fluorescent particles (50 particles/cell) were added to monocyte cultures for 2 h. Phagocytosis was measured by flow cytometry (A). Each data point represents the mean of a separate experiment, *n* = 4 per experiment (B). Average mean fluorescence intensity (MFI) values per experiment were assessed (C). Mann–Whitney tests were performed for nonparametric data. Unpaired Student’s *t*-tests were performed for parametric data. ns = nonsignificant.

Bone marrow progenitors, differentiated *in vitro*, showed no age-related defect in macrophage phagocytosis (Fig. 3). This suggests that myeloid progenitors are not intrinsically defective in phagocytosis. However, this artificial process is very different to *in vivo* differentiation, and the bone marrow environment in old mice could impact on macrophage function at the monocyte stage of development. To investigate this hypothesis, bone marrow-resident monocytes were isolated directly from the bone marrow of young and old mice. Fluorescent particles were added to monocytes from young and old mice for 2 h, and phagocytosis was assessed by flow cytometry. As expected, phagocytic efficiency in bone marrow monocytes was lower than both peritoneal macrophages and BMDMs. We observed considerable variability between levels of phagocytosis between experiments. Thus, data were pooled from four independent experiments, and no significant difference in phagocytosis was observed between bone marrow monocytes isolated from young and old mice (Fig. 4A,B). Furthermore, analysis of mean particles taken up per cell also showed no significant difference between young and old groups (Fig. 4C). These findings in bone marrow monocytes and BMDMs indicated that factors in the periphery might be responsible for the defect in peritoneal macrophage phagocytosis.

### Factors in the peritoneum of old mice suppress macrophage phagocytosis

**Figure 5:**
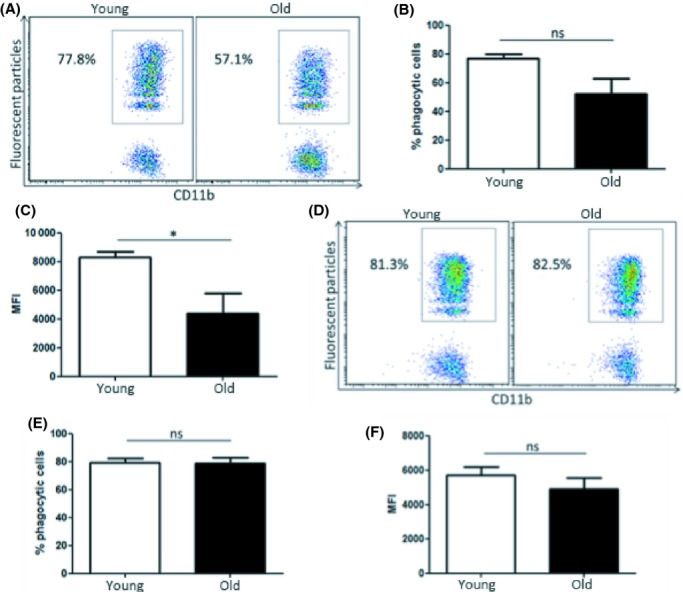
Factors in the peritoneum of old mice suppress macrophage phagocytosis. Peritoneal macrophages were isolated from young B6.SJL-*Ptprc*^*a*^ *Pepc*^*b*^/BoyJ mice and injected into the peritoneum of young and old C57BL/6 mice. Fluorescent particles were injected into the peritoneum of recipient mice 24 h later. Mice were euthanized 2 h later, and phagocytosis by CD45.1^+^ macrophages was assessed by flow cytometry. Percentage of phagocytic cells (A and B) and mean fluorescence intensity (MFI) values were analysed (C). Macrophages from young and old C57BL/6 were isolated and injected into the peritoneum of young B6.SJL-*Ptprc*^*a*^ *Pepc*^*b*^/BoyJ mice. Fluorescent particles were injected into the peritoneum of recipient mice 24 h later, and mice were euthanized after 2 h. Phagocytosis by CD45.2^+^ macrophages was assessed by flow cytometry. Percentage of phagocytic cells (D and E) and MFI values were analysed (F). *n* = 6 animals per group. Mann–Whitney tests performed for nonparametric data. Unpaired Student’s *t*-tests performed for parametric data. ns = nonsignificant. *P < 0.05

Phagocytosis by BMDMs and monocytes isolated from bone marrow was not significantly altered by aging. As such, we hypothesized that age-related impairments in phagocytosis may be a result of altered environmental signals in the periphery of old animals rather than cell-intrinsic defects in macrophages. Peritoneal macrophages consistently demonstrated an age-related impairment in phagocytosis in our experiments (Figs 1 and 2). Therefore, we hypothesized that microenvironmental factors in the peritoneum of old mice contribute to the observed age-related impairment in phagocytosis by peritoneal macrophages. To explore this hypothesis, an *in vivo* adoptive transfer experiment was designed. Briefly, purified peritoneal macrophages, expressing CD45.1, were isolated from young B6.SJL-*Ptprc*^*a*^ *Pepc*^*b*^/BoyJ mice (Fig. S2A). CD45.1^+^ macrophages were injected into the peritoneum of young and old C57BL/6 mice (Fig. S2B). C57BL/6 mice express CD45.2, which allowed the identification of CD45.1^+^ donor and host CD45.2^+^ macrophages. Fluorescent particles were injected into the peritoneum of these mice 24 h later. Mice were euthanized after 2 h, and phagocytosis of fluorescent particles by CD45.1^+^ donor macrophages was analysed by flow cytometry. In this setting, CD45.1^+^ macrophages from young mice were exposed to the peritoneal environment of old mice, and the impact on phagocytosis was assessed. CD45.1^+^ macrophages, injected into the peritoneum of old mice, showed a nonsignificant trend towards a reduction in percentage of phagocytic cells compared with those injected into young mice (Fig. 5A,B), and a statistically significant decrease between these groups was apparent when mean fluorescence intensity (MFI) values were analysed (Fig. 5C). This finding indicates that factors in the peritoneum of old mice inhibit macrophage phagocytosis. Further analysis of flow cytometry data confirmed that fluorescent particles were taken up by predominantly F4/80^+^ macrophages *in vivo* and not by other cell types in the peritoneum (Fig. S2C).

It is well established that macrophages adapt to signals in the local microenvironment. We hypothesized that the microenvironment in the peritoneum of young mice would increase the phagocytic capacity of macrophages from old mice. To test this hypothesis, macrophages from young and old C57BL/6 mice, expressing CD45.2, were isolated and transferred into the peritoneum of young B6.SJL-*Ptprc*^*a*^ *Pepc*^*b*^/BoyJ mice. Fluorescent particles were injected into the peritoneum of recipient mice 24 h later. Mice were euthanized 2 h later, and phagocytosis by CD45.2^+^ macrophages was assessed. Macrophages from old mice, transferred into the peritoneum of young mice, showed similar phagocytic efficiency as macrophages from young mice. This was evident in the analysis of both percentage of phagocytic cells (Fig. 5D,E) and MFI values (Fig. 5F). These data reinforce the central role of microenvironmental factors in dictating macrophage function within the peritoneum.

**Figure 6:**
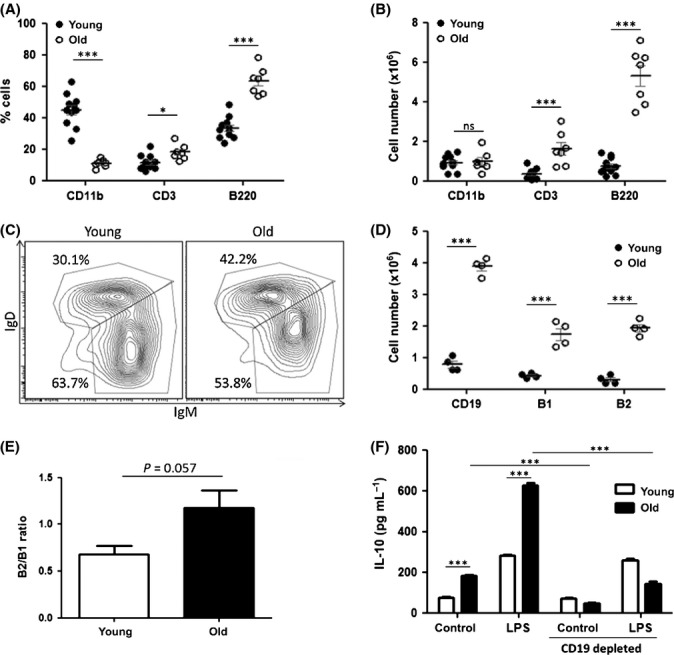
Aging results in significant changes in the cellular environment in the peritoneum of old mice. Percentages of peritoneal cells expressing CD11b, CD3 and B220 were assessed by flow cytometry in young and old mice (A). Cell counts were carried out, and absolute numbers of CD11b^+^, CD3^+^ and B220^+^ cells were calculated (B). Total peritoneal cells from young and old mice were stained for CD19, and CD19^+^ cells were assessed for expression of IgM and IgD (C). IgM^hi^IgD^lo^ cells were designated B1 cells and IgD^hi^IgM^lo^ cells were designated B2 cells. B1, B2 and CD19^+^ cell numbers were quantified in young and old mice (D). Proportions of B2 vs. B1 cells were calculated (E). Each data point represents one mouse. IL-10 production by total peritoneal cells, and CD19^+^-depleted peritoneal cells was assessed in both unstimulated and cultures stimulated with LPS (100 ng mL^−1^) for 24 h (F). One representative experiment of two separate experiments is shown. Mann–Whitney tests were performed on nonparametric data. Unpaired Student’s *t*-tests were performed on parametric data. ns = nonsignificant *P < 0.05 ***P < 0.001.

We then sought to identify factors within the peritoneum of old mice that could potentially impact the phagocytic capacity of macrophages. As we observed a reduction in the percentage of macrophages in the peritoneum of old mice (Fig. 1A), we next carried out immunophenotyping of the peritoneum in young and old mice. Total peritoneal cells were harvested from young and old mice, and absolute numbers and proportions of CD11b^+^, CD3^+^ and B220^+^ cells were assessed. As expected, the percentage of CD11b^+^ cells was reduced in old mice (Fig. 6A). Interestingly, there was no difference in absolute numbers of CD11b^+^ cells between young and old mice (Fig. 6B), which indicated an increase in other cell populations. Indeed, we found a significant increase in both the percentage and absolute cell number of CD3^+^ cells in old mice (Fig. 6A,B). Furthermore, we observed a striking increase in both the percentage and cell number of B220^+^ cells in old mice (Fig. 6A,B), demonstrating clear differences in the cellular environment in the peritoneum of old mice compared with young mice. B220 is frequently used as a B-cell marker; however, it can also be expressed on plasmacytoid dendritic cells and activated T cells (Renno *et al*., 1998; Ardavin, 2003). Therefore, to confirm an increase in B cells, peritoneal CD19^+^ cell numbers were also assessed. As expected, there was a significant increase in CD19^+^ cells in the peritoneum of old mice compared with young mice (Fig. 6D). Further investigation of B-cell subsets was carried out. Using an established gating strategy (Ghosn *et al*., 2010), CD19^+^ cells were divided into two populations, IgM^hi^IgD^lo^ and IgD^hi^IgM^lo^, representing B1 and B2 cells respectively. There was a significant increase in the number of both B1 and B2 cell subsets in the peritoneum of old mice compared with young mice (Fig. 6D). There was a trend towards higher proportions of B2 cells in the peritoneum of old mice, but this did not quite reach statistical significance (Fig. 6E; *P* = 0.057). These striking cellular differences are likely to result in changes in soluble factors in the peritoneum. Indeed, we observed that IL-10 production by total peritoneal cells was significantly increased, both in unstimulated conditions and when stimulated by LPS (Fig. 6F). In order to identify the source of IL-10, we depleted CD19^+^ cells from total peritoneal cells from young and old mice. IL-10 production by peritoneal cells from old mice was significantly reduced upon CD19^+^ depletion, both in resting conditions and following LPS stimulation, indicating that peritoneal B cells were responsible for the increased production of IL-10 in peritoneal cultures from old mice.

## Discussion

Phagocytosis by macrophages is a crucial process in immune defence against invading pathogens and also in tissue repair. Aging results in increased susceptibility to infection and impaired wound healing. As such, alterations in phagocytic capacity of macrophages will have consequences for the health of elderly individuals. We sought to investigate the impact of aging on phagocytosis by macrophages from young and old mice. ‘Old’ mice in this study were 15-to 20-month-old C57BL/6 male mice purchased from Charles River Laboratories and housed in a conventional unit. These mice were sourced from one colony throughout this study. It has been reported that the median lifespan of C57BL/6 male mice can be up to 28.5 months in a specific pathogen-free unit (Yuan *et al*., 2009). However, an increase in the incidence of tumours, skin conditions and other morbidities that would skew immune responses were frequently observed in mice over 20 months of age. In order to reduce confounding factors in our experiments, such as undetected pathology in old mice, we chose 20 months as a maximum age. Young mice in this study were 8–12 weeks old representing early adulthood and full immunological maturity.

In this study, peritoneal macrophage cultures were first used as a model system to investigate phagocytosis by macrophages from young and old mice. Many previous studies have relied on adherence to tissue culture plastic as a method to isolate peritoneal macrophages. However, this method introduces variability and can result in lower purity of macrophages. It has recently been shown that contamination of macrophage cultures with other cell types, such as eosinophils, NK cells or CD8^+^ T cells, can alter functional readouts, highlighting the importance of high purity in macrophage experiments (Schleicher *et al*., 2005; Misharin *et al*., 2012). In addition, we have shown that the percentage of macrophages in the peritoneum is decreased with age. As a result of this, the use of adherence as a method of purification could result in unequal numbers of macrophages in young and old conditions and potentially skew results, unless corrected for by protein assay or other normalization assays. This observation could account for some of the previously discussed variability reported in macrophage aging studies and also may have implications for the interpretation of some earlier studies where purification or absolute quantification was not carried out. Following pilot purification studies, a monocyte enrichment kit was selected, routinely resulting in >95% purity of CD11b^+^/F4/80^+^ double-positive cells. It is also important to note that because this study sought to investigate fundamental age-induced impairments in macrophage function, peritoneal macrophages were not elicited with thioglycollate, a process that results in alterations in macrophage populations, recruitment and function (Ghosn *et al*., 2010; Misharin *et al*., 2012). Therefore, our experiments have identified baseline impairments in macrophage function in the steady state.

Previous studies investigated the impact of aging on phagocytic capacity in macrophages using a range of experimental models and yielded some conflicting results (De La Fuente, 1985; Mancuso *et al*., 2001; Aprahamian *et al*., 2008). In our studies, we observed a significant and consistent decrease in phagocytosis by peritoneal macrophages from old mice compared with young mice, and this finding was confirmed *in vivo*. There are many different factors in macrophage experiments that could result in conflicting data, including species, strain, housing conditions, macrophage subsets, macrophage purification methods, quantity and quality of phagocytic target and incubation time. Studies in humans have also proven to be challenging considering the genetic and immune status variability in the human population and the difficulty in obtaining tissue macrophages (Fietta *et al*., 1994; Hearps *et al*., 2012).

The ultimate goal of research in immune aging is to aid the development of treatments that will enhance immunity and thus increase the quality of life in the elderly population. In order to modify immune function, it is essential to understand the mechanisms underlying age-related functional impairments. It was unclear from our initial studies whether age-related impairments were intrinsic to macrophages or the result of cell-extrinsic factors in old animals. To investigate this, we carried out phagocytosis assays using BMDMs, a commonly used experimental model of macrophage biology. In these experiments, bone marrow was harvested and progenitors were differentiated *in vitro*, in the absence of environmental factors that would be present in old mice. Interestingly, BMDMs from old mice showed no age-related decrease in phagocytosis of fluorescent particles. These results suggested no intrinsic defect in phagocytosis in myeloid progenitors from old mice. Related findings have been reported in at least two other studies. For example, aging altered macrophage polarization in splenic macrophages but not in BMDMs (Mahbub *et al*., 2012). In addition, Chen *et al*. observed enhanced NO production in response to LPS in peritoneal macrophages from old mice but not in BMDMs (Chen *et al*., 1996). These studies were carried out in BALB/c and CBA/CA mouse strains, respectively, demonstrating that this finding was consistent across different strains.

A possible explanation for our findings in BMDMs is that the aging bone marrow microenvironment causes cellular defects during differentiation, but not at the early progenitor stage. To investigate this, bone marrow-resident monocytes, which had developed *in vivo*, were isolated directly from bone marrow of young and old mice. However, monocytes from young and old mice showed similar levels of phagocytosis, indicating that the environment in bone marrow in old mice did not cause significant cellular defects in phagocytic capacity in monocytes/macrophages. These data suggested that factors in the periphery of old mice could be responsible for age-related impairments in peritoneal macrophage phagocytosis.

It is also important to note that recent studies have questioned the contribution of monocytes in the maintenance of peritoneal macrophages in basal conditions (Yona *et al*., 2013). It has been suggested that peritoneal macrophages are maintained by local proliferation during homoeostasis. In this case, long-term exposure to factors in the peritoneum of old mice could play an important role in shaping the phenotype of these self-renewing cells. Similarly, Stout and Suttles hypothesized that age-associated dysfunction in macrophages is due to functional adaption to the age-associated changes in tissue environments (Stout & Suttles, 2005), which was also recently discussed by Mahbub *et al*. (2012). We designed an *in vivo* experiment to further explore this hypothesis. Macrophages from young mice were injected into the peritoneum of old mice and a phagocytosis assay was carried out *in vivo* in the presence of environmental factors in the old peritoneum. Indeed, when we assessed mean particle uptake using MFI values, we observed that the peritoneal environment in old mice downregulated phagocytosis by macrophages from young mice. This indicates that macrophage-extrinsic factors in the peritoneum are responsible for the impairment in phagocytic capacity of peritoneal macrophages in old mice. Furthermore, when macrophages from old mice were injected into the peritoneum of young mice, these cells had similar phagocytic capacity as macrophages from young mice injected into young recipients. This suggests that no irreversible intrinsic defects were present in macrophages from the peritoneum of old mice and factors in the peritoneal environment of young mice supporting effective phagocytosis. These findings support the hypothesis that environmental factors play a vital role in age-related changes in peritoneal macrophage phagocytosis.

We investigated the cellular environment in the peritoneal cavity of old mice to identify factors that could impact macrophage phagocytosis. Immunophenotyping of peritoneal cellular subsets demonstrated striking differences between young and old mice. Interestingly, the B220^+^ cell population showed the most significant expansion, which was confirmed to be a B-cell population by CD19 expression. Increased recruitment and/or expansion of B cells in the peritoneal cavity could underlie increased B cells in aged mice. To investigate possible differences in B-cell recruitment, freeze-dried and minimally reconstituted peritoneal lavages of young and old mice were assayed for the B-cell chemokine CXCL13. Results demonstrated a trend towards increased CXCL13 in lavages from old mice; however, this did not reach statistical significance (data not shown). While this does not rule out the possibility of enhanced recruitment, it is also reasonable to hypothesize that increased peritoneal B cells in old mice may arise from cells resident in the peritoneum. Indeed, Stall *et al*. reported an increase in B cells in old mice as a result of expansion of self-renewing cells in the peritoneum (Stall *et al*., 1988). More recently, Hao *et al*. investigated the presence of a unique population, termed age-associated B cells (ABCs), in old mice. Interestingly, this study concluded that ABCs were derived from exhausted mature B cells (Hao *et al*., 2011).

The observed age-related increase in peritoneal B cells could potentially have an impact on macrophage function. It has been reported that peritoneal B cells have the potential to regulate macrophage phenotype (Wong *et al*., 2010), and Popi *et al*. demonstrated that B cells in the peritoneum can impair phagocytosis via IL-10 production (Popi *et al*., 2004). Indeed, we also observed that there was a significantly increased IL-10 production by total peritoneal cells from old mice, both in the resting state and when activated with LPS, which was lost upon depletion of B cells in both cases.

In conclusion, we have demonstrated that aging results in impaired phagocytosis by peritoneal macrophages. These findings have implications for defence against microbial infection and also tissue repair in the elderly. Phagocytosis by BMDMs and bone marrow-resident monocytes was unaffected by age, indicating no defect in phagocytosis in the myeloid progenitors of old mice in our model. Our findings indicate that environmental factors in the peritoneum of old mice are responsible for the observed age-related impairment in peritoneal macrophage phagocytosis. Importantly, this suggests that age-related impairments in macrophage phagocytosis could be reversible and as such could be targeted therapeutically, either directly or by modifying the tissue microenvironment. As such, tissue-specific pathologies could be targeted independently of systemic effects, thereby minimizing adverse events associated with systemic immunomodulatory therapies.

## Experimental procedures

### Animals

‘Young’ and ‘old’ C57BL/6 mice (8–12 weeks and 15–20 months, respectively) were bred in-house or purchased from Charles River Laboratories and acclimatized in common environments for a minimum of 4 weeks. B6.SJL-*Ptprc*^*a*^ *Pepc*^*b*^/BoyJ mice, expressing CD45.1, were purchased from the Jackson Laboratory and bred in-house. All animal maintenance and experiments were in compliance with the UK Home Office and approved by the Queen’s University Ethical Review Committee.

### Macrophage preparation and culture

#### Peritoneal macrophages

Peritoneal cells were harvested by peritoneal lavage with 8–10 mL of ice-cold PBS. Peritoneal macrophages were purified using a monocyte enrichment kit (StemCell Technologies, Grenoble, France) as per manufacturer’s instructions. The resulting cell population was >95% CD11b^+^/F4/80^+^ double positive. Peritoneal macrophages were plated at 5 × 10^5^ cells mL^−1^ in DMEM supplemented with FCS (10%), L-glutamine (2 mm) and penicillin/streptomycin (100 U mL^−1^, 100 μg mL^−1^) (all purchased from PAA Laboratories, Somerset, UK). Peritoneal macrophages were cultured overnight prior to phagocytosis assays.

#### Bone marrow-derived macrophages (BMDMs)

Bone marrow was flushed from femurs and tibiae of C57BL/6 mice. Bone marrow cells were cultured for 7 days in DMEM supplemented with FCS (10%), L-glutamine (2 mm), penicillin/streptomycin (100 U mL^−1^, 100 μg mL^−1^) and 15% conditioned medium from the L929 fibroblast cell line. After 7 days, cultures contained >95% macrophages as assessed by CD11b and F4/80 staining. BMDMs were plated at 1 × 10^6^ cell mL^−1^ for subsequent experiments in 48-well tissue-culture-treated plates. BMDMs were cultured overnight prior to phagocytosis assays.

#### Bone marrow-resident monocytes

Bone marrow was prepared as described above. Monocytes/macrophages were isolated from bone marrow cells using a monocyte enrichment kit (StemCell Technologies). Purity was assessed by CD11b^+^/Ly6C^+^ cells and found to be >85%. Bone marrow monocytes were cultured for 2 h prior to phagocytosis assays.

### *In vitro* phagocytosis assay

Macrophage/monocyte cultures were incubated with fluorescent particles (50 particles/cell unless otherwise stated) (Spherotech, Lake Forest, IL, USA) for 2 h. Cells were then harvested and analysed by flow cytometry. Briefly, cells were incubated with anti-CD16/CD32 (eBioscience, Hertfordshire, UK) to block Fc receptors. Cells were then stained with antibodies to CD11b (eBioscience) and F4/80 (Biolegend, London, UK) and fixed with Medium A (Life Technologies, Paisley, UK) prior to acquisition on a flow cytometer (BDFACSCantoII, Becton Dickson, Oxford, UK). Analysis of flow cytometry data was carried out using flowjo software (Treestar, Ashland, OR, USA). Viable cells were selected based on FSC vs. SSC. Single cells were selected using FSC-W vs. FSC-A. Macrophages were identified based on CD11b and F4/80 expression.

### *In vivo* phagocytosis assay

Fluorescent particles (Spherotech) were diluted in sterile saline and injected into the peritoneum of C57BL/6 mice. Mice were euthanized after 2 h, and peritoneal cells were harvested as described above. Total peritoneal cells were stained with antibodies for CD11b (eBioscience) and F4/80 (BioLegend) and analysed by flow cytometry.

### Adoptive transfer of CD45.1^+^ macrophages

Peritoneal macrophages, expressing CD45.1, were purified from young male B6.SJL-*Ptprc*^*a*^ *Pepc*^*b*^/BoyJ mice using a monocyte enrichment kit. Peritoneal macrophages (1 × 10^6^ cells/mouse) were injected into the peritoneum of young and old C57BL/6 mice. Fluorescent particles were also injected into the peritoneum of these mice 24 h later. Mice were euthanized after 2 h, and peritoneal cells were harvested. Total peritoneal cells were stained with antibodies for CD45.1, CD11b (eBioscience) and F4/80 (BioLegend) and subsequently analysed by flow cytometry. Alternatively, macrophages were harvested from young and old C57BL/6 mice and injected into the peritoneum of young B6.SJL-*Ptprc*^*a*^ *Pepc*^*b*^/BoyJ mice, and the experiment carried out as described above.

### Immunophenotyping of peritoneal cells

Total peritoneal cells were harvested from young and old mice. Cells were prepared for flow cytometry as described above. CD11b, B220, CD3 and CD19 antibodies were all purchased from eBioscience.

### B-cell depletion

CD19^+^ cells were depleted from total peritoneal cells from young and old mice using a Mouse CD19 Positive Selection Kit (StemCell Technologies).

### Immunofluorescence microscopy

Macrophages were seeded (70 000 cells/well) and allowed to adhere for approximately 3 h. Fluorescent particles were added for 2 h. For the assessment of *in vivo* phagocytosis, peritoneal cells were isolated after a 2 h-incubation period (see above) and immediately adhered to chamber slides for approximately 3 h. Media were removed and slides were washed with PBS. Cells were fixed with 4% paraformaldehyde, washed and blocked with 10% normal goat serum (Vector laboratories, Peterborough, UK). Cells were stained with CD11b-FITC (eBioscience), washed and stained with DAPI (300 nm, Life Technologies). Finally, slides were washed, mounted with ProLong Gold (Life Technologies) and visualized with an epifluorescence microscope (Leica DM5500, Milton Keynes, UK).

### IL-10 quantification

Supernatants were collected from total peritoneal cell cultures and assayed for IL-10 using a mouse IL-10 Duoset kit (R&D, Abingdon, UK) as per manufacturers’ instructions.

### Statistics

Data were tested for statistical significance using unpaired, two-tailed Student’s *t*-tests for parametric data. Mann–Whitney tests were used for nonparametric data such as percentages. anova followed by Bonferroni test were performed for Fig. 6F.

## Supporting Information

**Fig. S1** Immunomagnetic purification of peritoneal macrophages and gating strategy for analysis of highly phagocytic cells. **Fig. S2** Identification of adoptively-transferred macrophages and verification that fluorescent particles were predominantly taken up by F4/80^+^ macrophages.
