# Supplementary material for: Aging impairs peritoneal but not bone marrow-derived macrophage phagocytosis
Source: Aging Cell. 2014 May 12;13(4):699–708. doi: 10.1111/acel.12223 (PMC4326936; doi:10.1111/acel.12223)

**SUPPLEMETARY FIGURE 1.** Immunomagnetic purification of peritoneal macrophages and gating strategy for analysis of highly phagocytic cells.


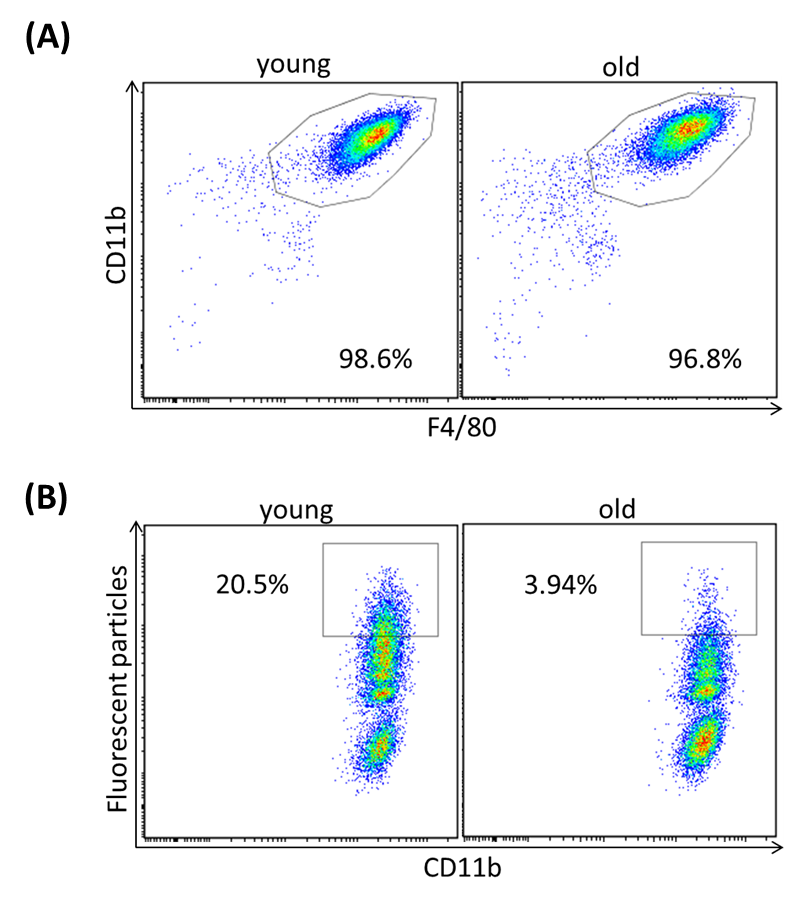


**SUPPLEMETARY FIGURE 2.** Identification of adoptively-transferred macrophages and verification that fluorescent particles were predominantly taken up by F4/80^+^ macrophages.


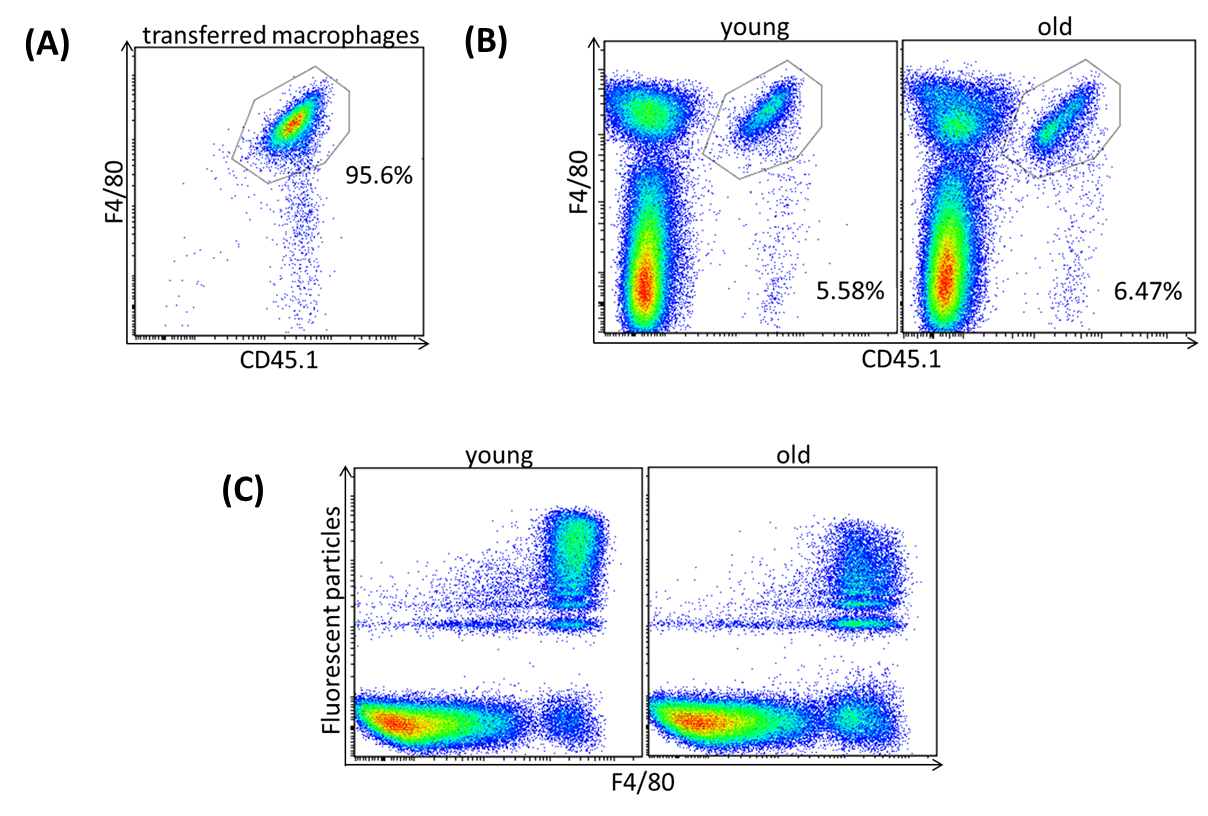

Supplement: Supplementary file 1 — Fig. S1 Immunomagnetic purification of peritoneal macrophages and gating strategy for analysis of highly phagocytic cells. Fig. S2 Identification of adoptively-transferred macrophages and verification that fluorescent particles were predominantly taken up by F4/80+ macrophages. [file acel0013-0699-sd1.docx]
